# Giant Accessory Right-Sided Suprarenal Spleen in Thalassaemia 

**DOI:** 10.1155/2013/269543

**Published:** 2013-02-25

**Authors:** A. Arra, Michael J. Ramdass, A. Mohammed, O. Okoye, D. Thomas, S. Barrow

**Affiliations:** Department of Surgery, University of the West Indies, General Hospital, Port-of-Spain, Trinidad and Tobago

## Abstract

An accessory spleen is defined as ectopic splenic tissue that develops due to failure of fusion of cells during embryonic development as they migrate from the midline to the left upper quadrant. While benign, complications may arise which include trauma, torsion, or infarction of the ectopic tissue. Additionally, patients who have had a splenectomy secondary to treatment for previous pathology such as a haematological malignancy or idiopathic thrombocytopenia purpura may experience persistent symptoms due to the accessory splenic tissue. The presence of an accessory spleen is therefore of significant diagnostic and therapeutic importance. To the best of the authors' knowledge, this case is the second and largest reported case of a giant right suprarenal accessory spleen and highlights the difficulty in differentiation of these masses from malignant adrenal tumours.

## 1. Introduction

The spleen is an organ found in the left hypochondrial region of the abdomen, between the gastric fundus and left hemidiaphragm. It plays an important role in the haematological and immune systems and acts as a reservoir for approximately 10–20% of blood volume [1].

Congenital abnormalities of the spleen are rare, but include splenic agenesis, polysplenia, and the presence of accessory spleens or splenunculi [2]. Accessory spleens are thought to be present in about 30% of the population, but go widely unrecognized due to their small size. They may be identified in cases of torsion or rupture, at postmortem, or as an incidental finding during radiological investigation for other conditions [3].

This case is the second and largest reported case of a giant right suprarenal accessory spleen and highlights the difficulty in differentiation of these masses from malignant adrenal tumours.

## 2. Case Report

A 24-year old male thalassaemic patient presented electively with a history of a right-sided abdominal mass on self-examination, with no other complaints. A magnetic resonance image of the abdomen was requested, which revealed a 20 cm mass arising in the right suprarenal region, which was suspected to be a malignant adrenal tumour. Investigations revealed no evidence of adrenal dysfunction. The Patient was optimized for surgery in keeping with haematological recommendations and prepared and consented for removal of the right suprarenal mass.

A Chevron incision was made to provide adequate access. At surgery, a giant highly vascularized tumour was excised from the right suprarenal region. There was an easily dissectible plane around the mass and its blood supply originated from a short pedicle from the aorta and inferior vena cava. There was no evidence of invasion into the surrounding structures. The patients' primary spleen and other intra-abdominal organs were otherwise normal in anatomy and size.

**Figure 1:**
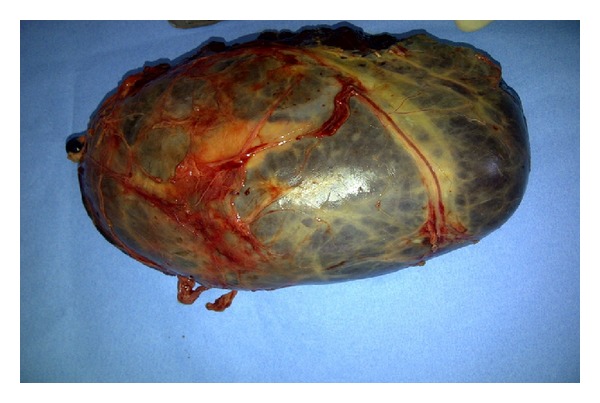
A 20 cm right suprarenal accessory spleen removed.

**Figure 2:**
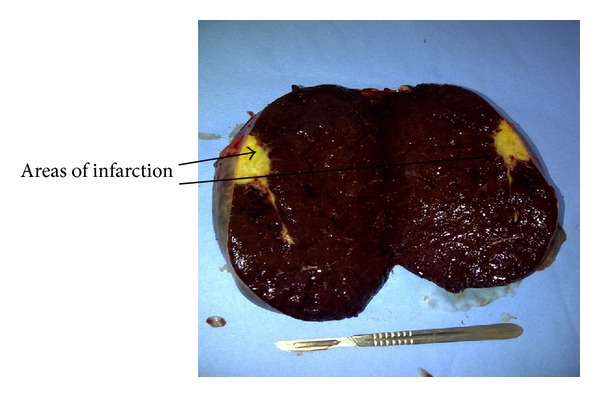
Cut specimen of massive accessory spleen showing mostly normal splenic tissue with some yellow areas of infarction.

**Figure 3:**
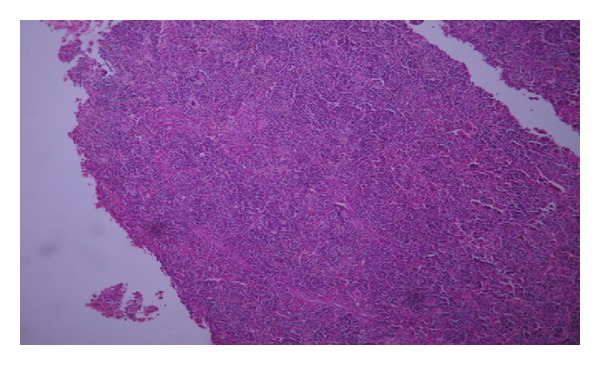
Histology showing normal splenic tissue (low power).

**Figure 4:**
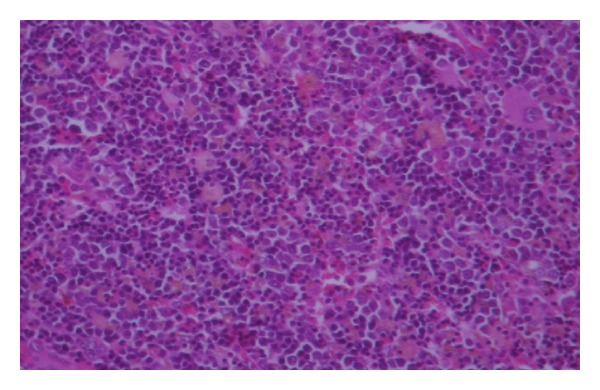
Histology showing normal splenic tissue (high power).

Histological examination revealed this mass to be normal splenic tissue with a small central area of infarction and no evidence of malignancy noted (Figures 1, 2, 3, and 4).

## 3. Discussion

An accessory spleen may develop during embryological development due to failure of lobulation of splenic tissue to fuse as the spleen migrates from the midline to its position in the left upper quadrant. They may occur anywhere along this path in the abdomen including the hilum of the spleen, the tail of the pancreas, the gastrosplenic and splenorenal ligaments, the walls of the stomach or intestines, the greater omentum, the mesentery, the adrenals, and the gonads in the case of splenogonadal fusion and do not usually exceed 2-3 cm in size [1].

Satellite splenic tissue can also occur in splenosis, which is a condition whereby fragments of splenic tissue are implanted at well-vascularized areas of the abdominal cavity following splenectomy or rupture of the spleen [4].

Enlargement of the spleen can result due to a number of conditions such as B-lymphoproliferative disorders, haematological disorders, and hyperreactive malarial splenomegaly [5] as well as portal hypertension [6]. This particular patient was thalassaemic, which is characterized by the production of abnormal haemoglobin with a chronic haemolytic anaemia. Splenomegaly occurs in such patients mainly due to excessive red cell destruction and extramedullary haemopoiesis as well as secondary to iron overload that develops from multiple lifelong blood transfusions [7].

There have been previous cases that describe an accessory spleen mimicking a left-sided adrenal tumour [1, 8–10]; however, it is exceedingly rare to discover an ectopic accessory spleen in the right suprarenal region with only one such case reported by Kim et al. in 2008. This was reported in a 68-year-old man with an incidentally detected retroperitoneal mass. The computed tomography scan of the abdomen revealed the presence of a retroperitoneal tumor (4.0 × 3.8 cm) at the right suprarenal space. Laparoscopic excision was carried out and histological examination confirmed the mass to be splenic tissue [11].

The diagnosis of an accessory spleen should be considered as a differential for retroperitoneal and intraabdominal masses despite position. This case, to the best of the authors' knowledge, is the largest right suprarenal accessory spleen reported, measuring approximately 20 cm in size, and should be considered as a differential in planning an approach to surgery especially in patients with underlying haematological or lymphoproliferative pathology.
